# Clinical approach to palpitations in pregnancy

**DOI:** 10.1016/j.clinme.2024.100276

**Published:** 2024-12-16

**Authors:** Ferha Saeed, Kirun Gunganah, Anna S. Herrey

**Affiliations:** a Department of Obstetrics and Gynaecology, Newham University Hospital, Barts Health NHS Trust, Honorary Clinical Senior Lecturer, Barts & The London School of Medicine & Dentistry, Queen Mary University of London, London, UK; b Department of Diabetes and Endocrinology, Newham University Hospital, Barts Health NHS Trust, London, UK; c Department of Cardiology, Barts Heart Centre and Newham University Hospital, Barts Health NHS Trust, Honorary Senior Lecturer, William Harvey Research Institute, Queen Mary University of London, London, UK

**Keywords:** Pregnancy, Arrhythmia, Investigation, Treatment

## Abstract

Palpitations are common in pregnancy and warrant investigation. Palpitations may be caused by non-cardiac and cardiac causes. Patients with structural or functional abnormalities or inherited cardiovascular disease are more likely to develop arrhythmia, especially during pregnancy when the mother’s body undergoes extensive physiological adaptations, which further contribute to an increased arrhythmia risk. While isolated ectopic beats do not require treatment, some heart rhythm disturbances can be life-threatening for mother and baby and mandate prompt intervention.

Haemodynamically unstable patients should be electrically cardioverted. If the patient is stable, medical management is indicated, and early involvement of the pregnancy heart team can help facilitate appropriate treatment. In complex arrhythmia, consultation of an arrhythmia expert should be sought . Many anti-arrhythmics are safe in pregnancy, and it is important to reassure the pregnant patient of this.

## Introduction

Palpitations are commonly experienced during pregnancy.^1^ While often benign, palpitations can be a source of concern for the pregnant woman and her clinician. The frequency of pregnancies complicated by arrhythmia is increasing.^2^ In the western world, 1–4% of pregnancies are affected by maternal cardiac disease,^3^ with cardiovascular disease remaining a leading cause of death in pregnant and recently postpartum women in the UK.^4^ Pre-existing cardiac disease predisposes to the development of heart rhythm disturbances in pregnancy and the postpartum period. This may be related to structural abnormalities, for example in patients with valve disease, congenital heart disease (corrected and uncorrected), impaired left or right ventricular function, or because of an intrinsic predisposition to arrhythmia, such as long QT or Brugada syndromes.

This article outlines a practical approach to the evaluation and investigation of palpitations for the general physician, and briefly touches on treatment options of arrhythmia in pregnancy.

## Why are palpitations more common in pregnancy?

During pregnancy, the body undergoes reversible circulatory and cardiac adaptations to accommodate the growing fetus. These changes predispose to palpitations and arrhythmia in the following ways.

Cardiac output rises by 30–50% due to a 50% increase in blood volume and a 15% rise in resting heart rate. Systemic and pulmonary vascular resistance decrease by 35–40%.^5^ The effects of pregnancy hormones on the myocardium, stretch caused by hypervolaemia leading to electrical instability, as well as increased levels of circulating catecholamines all contribute to creating a pro-arrhythmic state.^6^ Dilutional anaemia occurs as a result of plasma volume expansion. This can further increase heart rate, perceived as palpitations, with higher visceral awareness in pregnancy.^7^

## Clinical presentation

Palpitations are most commonly described as a fluttering, pounding or racing sensation in the chest, skipped beats or an irregular rhythm. Important factors are onset and duration. Associated symptoms can include dizziness, dyspnoea, chest discomfort or syncope. Palpitations may occur at rest, during physical activity, or in response to emotional stress. Certain positions, such as lying down, can exacerbate the sensation. Triggers can include caffeine, nicotine or even dehydration. Palpitations may be linked to anaemia, electrolyte abnormalities or thyroid dysfunction, which are not uncommon in pregnancy.

The distinction of normal symptoms of pregnancy from symptoms indicating pathology can be difficult. The following red flags^8^ warrant further evaluation:

1. (pre-)Syncope with palpitations can indicate serious arrhythmia or haemodynamic instability.
2. Chest pain may suggest ischaemic heart disease, particularly in women with risk factors such as hypertension or diabetes.
3. Shortness of breath may be indicative of heart failure, pulmonary embolism or significant arrhythmias.
4. Irregular heartbeat, especially if sustained, may indicate atrial fibrillation, flutter or other potentially serious arrhythmia.
5. History of structural heart disease: women with a history of congenital heart disease, cardiomyopathy, valvular disease or previous myocardial infarction are at higher risk for serious complications during pregnancy and should be closely monitored. Although rare, ventricular tachycardia (VT) in pregnancy most commonly occurs in women with underlying cardiomyopathy.^9^
6. Family history of sudden cardiac death: unexplained death at a young age could indicate inherited arrhythmia or cardiomyopathy.

## Investigations

Not all palpitations are caused by underlying arrhythmia, and not all arrhythmia requires treatment, but all pregnant patients with palpitations should be investigated. The first step includes history and examination, relevant blood tests, a 12-lead electrocardiogram (ECG), and a 24-hour Holter monitor. An echocardiogram should be considered if arrhythmia or high ectopic burden are detected, or there is a suspicion of structural heart disease. Impaired left ventricular (LV) function is known to be a predictor of maternal cardiac events, including arrhythmia.^10^

### Blood tests

A full blood count (FBC) should be carried out as anaemia can increase heart rate and cause palpitations. Urea and electrolytes should also be conducted as electrolyte abnormalities, such as disturbances in potassium, magnesium and calcium, can present with palpitations. Thyroid function tests should also be done as thyroid disorders, in particular thyrotoxicosis, are well recognised to cause palpitations. It is important to remember that thyroid function has different trimester-specific ranges in pregnancy.^11^

### 12-lead ECG

Even in the absence of ongoing arrhythmia, the 12-lead ECG can yield important information, such as evidence of pre-excitation, unusual QRS morphology eg Brugada, or long QT. In advanced pregnancy, however, the ECG may show pregnancy-related changes such as T-wave inversion, left axis deviation or ST depression.^12^

### 24-hour ECG

Often the first step in the assessment of palpitations, a 24-hour ECG can capture abnormal heart rhythms associated with cardiac symptoms. It is useful for assessing heart rate variability to help distinguish atrial tachycardia from a physiological heart rate increase. In patients who are prone to arrhythmia (eg those with structural heart disease), it can detect asymptomatic arrhythmia.

### Single channel ECG monitoring and implantable loop recorders

If palpitations occur infrequently, single channel ECG monitors can be worn for up to 2 weeks. If the level of suspicion is high for rare but significant arrhythmia, loop recorders can be implanted during pregnancy.

### Echocardiography

Transthoracic echocardiography is the ‘go-to’ imaging modality used for structural or functional heart disease, bearing in mind that physiological adaptations of pregnancy will be reflected in echocardiographic findings, eg increased cardiac chamber sizes, flow velocities and gradients. As pregnancy advances, the acoustic windows deteriorate due to cardiac displacement by the gravid uterus. Transoesophageal and stress echocardiography are rarely necessary.

### Cross-sectional imaging

When heart muscle disease is suspected, cardiac magnetic resonance (CMR) can safely be undertaken.^13^ Cardiac computed tomography (CT) may rarely be appropriate in selected cases of arrhythmia presenting in pregnancy.

### Electrophysiological (EP) studies

In selected circumstances EP studies can be used to not only investigate, but also treat incessant and/or potentially life-threatening arrhythmia when conservative options are ineffective (see below).

## Treatment

Isolated ectopics, bigeminy and trigeminy (both atrial and ventricular) are common and, in the context of a structurally and functionally normal heart, require no intervention. However, a high ectopic burden can result in reduced LV function.^14^, ^15^, ^16^ Many anti-arrhythmics are safe in pregnancy, and it is important to reassure the pregnant patient of this.^17^ Once arrhythmia has been detected, the pregnancy heart team should be involved in any decision making regarding the need for and the type of treatment.

### Supraventricular tachycardia

This is the most commonly encountered arrhythmia. Acutely, if the patient is haemodynamically unstable, electrical cardioversion should be used. Otherwise, vagal manoeuvres and adenosine can be tried, and if not effective, beta-blockers and flecainide may be used, which can be continued for maintenance throughout the pregnancy. Verapamil and digoxin can be used as second line except in patients with known pre-excitation. In difficult-to-treat atrial arrhythmia, an electrophysiologist should be involved at an early stage.

### Atrial fibrillation/flutter

In haemodynamically unstable atrial fibrillation/flutter (AF), prompt electrical cardioversion is necessary. If stable, beta-blockers are first-line treatment, digoxin and verapamil may be used except in patients with pre-excitation.^18^ Limited evidence exists on anticoagulation in patients with structurally normal hearts, despite pregnancy-related hypercoagulability. In the context of structural abnormalities, anticoagulation is often required. If cardioversion is considered for a stable patient, the general consensus is that no anticoagulation is required if AF has been present for less than 48 h, although a clear onset cannot always be established, and no guidelines exist with regard to pregnancy. If AF has been present for more than 48 h, the patient should be anticoagulated for a minimum of 4 weeks, or the presence of thrombus in the left atrial appendage excluded by transoesophageal echocardiography.^19^ Flecainide, beta-blockers and sotalol can be given for maintenance of sinus rhythm.

### Ventricular arrhythmia

Sustained ventricular arrhythmia in an unstable patient requires synchronised electrical cardioversion.^20^ If stable, beta-blockers, lignocaine and sometimes verapamil can be considered.^21^ Treatment options may vary depending on the aetiology, such as idiopathic, inherited and structural causes of VT,^6^ and an arrhythmia specialist should be consulted.

Although ablation is preferably deferred until postpartum, for refractory incessant and/or life-threatening tachyarrhythmia, catheter ablation using minimal or no fluoroscopy is feasible in pregnancy.^22^

## Key points

1) Palpitations are common in pregnancy and do not always indicate underlying arrhythmia however should be investigated.
2) Diagnosis is the first step in treating arrhythmia in pregnancy, and most tests can safely be undertaken where indicated.
3) In haemodynamically unstable tachyarrhythmia, prompt electrical cardioversion should be used.
4) Many anti-arrhythmics are safe for mother and baby.
5) Involve the pregnancy heart team early.

## Funding

This research did not receive any specific grant from funding agencies in the public, commercial or not-for-profit sectors.

## CRediT authorship contribution statement

**Ferha Saeed:** Writing – review & editing, Writing – original draft. **Kirun Gunganah:** Writing – review & editing, Writing – original draft. **Anna S. Herrey:** Writing – review & editing, Writing – original draft, Supervision, Conceptualization.

## Declaration of competing interest

The authors declare that they have no known competing financial interests or personal relationships that could have appeared to influence the work reported in this paper.
